# Immature characteristics of the East Anatolian Fault Zone from SAR, GNSS and strong motion data of the 2023 Türkiye–Syria earthquake doublet

**DOI:** 10.1038/s41598-024-61326-6

**Published:** 2024-05-09

**Authors:** Jiao Liu, Chuanchao Huang, Guohong Zhang, Xinjian Shan, Andrey Korzhenkov, Tuncay Taymaz

**Affiliations:** 1 State Key Laboratory of Earthquake Dynamics, Institute of Geology, China Earthquake Administration, Beijing, 100029 China; 2 Institute of Disaster Prevention, Sanhe, 065201 China; 3 Urumqi Institute of Central Asia Earthquake, China Earthquake Administration, Urumqi, 830011 China; 4 Institute of Physics of the Earth, Russian Academy of Sciences, Moscow, 123242 Russia; 5 Department of Geophysical Engineering, The Faculty of Mines, İstanbul Technical University, Ayazağa Campus, Maslak, Sarıyer, İstanbul, 34469 Türkiye; 6 School of Geosciences and Info-Physics, Central South University, Changsha, 410000 China

**Keywords:** Natural hazards, Solid Earth sciences

## Abstract

On February 6, 2023, an Mw 7.9 earthquake occurred in the western section of the East Anatolia Fault Zone (EAFZ). It was subsequently followed by an Mw 7.7 earthquake on the northern branch of the EAFZ, known as the Sürgü Fault Zone. Coseismic deformation fields were derived for these earthquakes using joint evaluation of near-field strong motion data, Global Navigation Satellite System data, and Synthetic Aperture Radar datasets. The coseismic slip distribution model was determined through the joint kinematic finite fault inversion. The Mw 7.9 earthquake was a left-lateral strike-slip event, predominantly occurring at depths up to 20 km. The earthquake displayed three distinct asperities that correlate well with bends and stepovers along the EAFZ. The Mw 7.7 earthquake also exhibited left-lateral strike-slip characteristics, with a major asperity along the Çardak Fault featuring a maximum slip of approximately 9.5 m at depths between 0 and 24 km. The occurrence of this unanticipated large Mw 7.9 catastrophic seismic event on a fault with low-intermediate structural maturity is noteworthy. In the vicinity of immature faults with multiple jogs, stress tends to accumulate at barrier locations. When the accumulated stress near several adjacent barriers reaches a certain threshold, it may result in the transformation of multiple barriers into asperities, triggering cascading ruptures.

## Introduction

**Figure 1:**
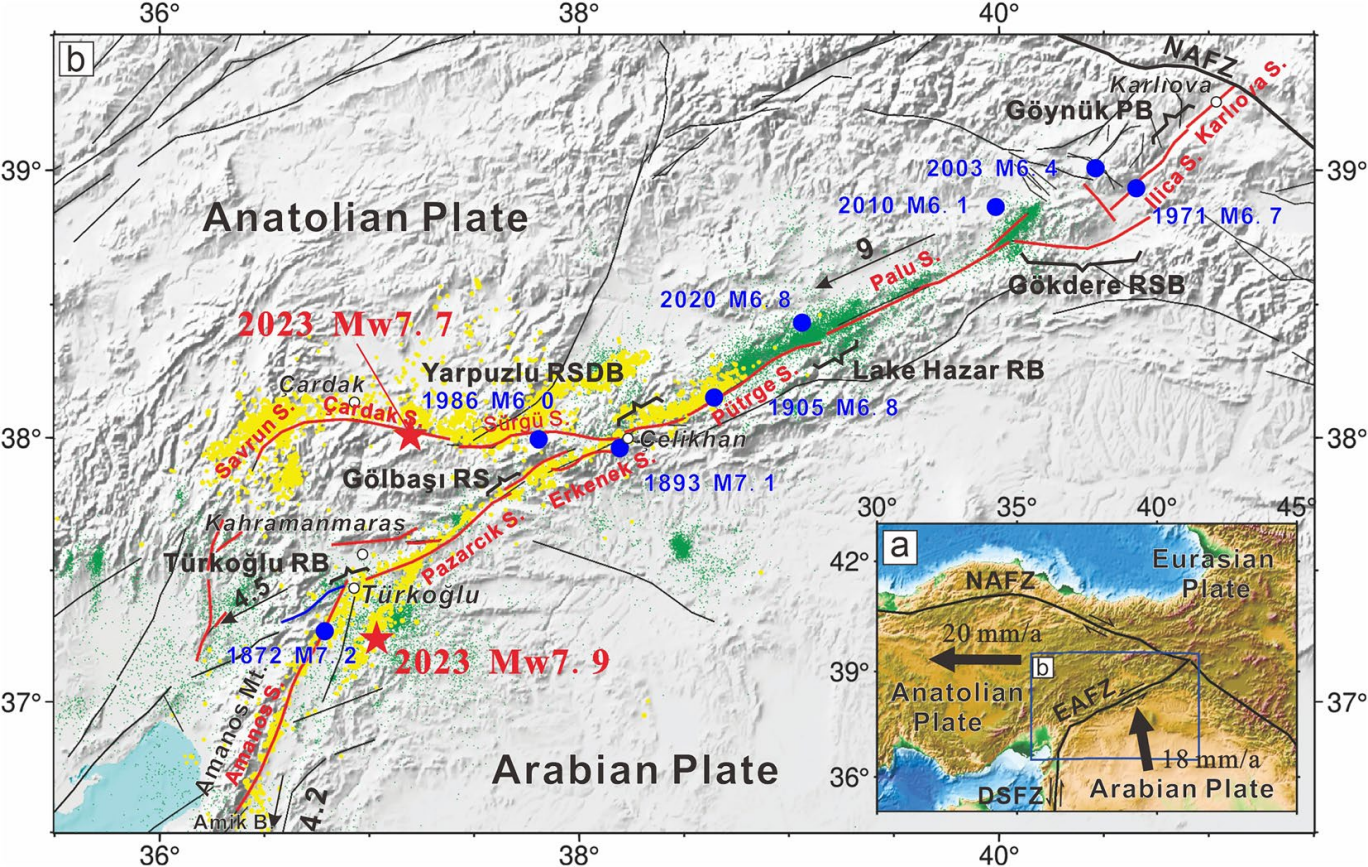
Tectonic setting of the EAFZ. (**a**) Schematic diagram of regional tectonic movement. Black arrows indicate plate motion and the blue rectangle represents the study area of the EAFZ. (**b**) Map of the EAFZ. Red lines represent the EAFZ, the blue line represents the buried fault referred to as fault-1, and the black lines represent other faults (modified after Duman and Emre^16^). Black brackets indicate bends or stepovers. RB: releasing bend; RS: releasing stepover; RSDB: restraining double bend; RSB: restraining bend; PB: paired bend. Blue solid circles represent the epicenters of moderate–large earthquakes over the past 200 years. Red stars represent the epicenters of the Mw 7.9 and Mw 7.7 earthquakes, respectively. Green dots represent epicenters of earthquakes between 2007 and 2020^13^. Yellow dots show the aftershocks of the 2023 Türkiye–Syria earthquake doublet.

The eastern Mediterranean segment of the Alpine–Himalayan orogenic belt, where the Eurasian Plate, Arabian Plate, and Anatolian Plate converge, is one of the most tectonically active regions in the world^1^. Compared with the stable Eurasian Plate, the Arabian Plate is moving northwestward at 18 mm/yr and the Anatolian Plate is escaping westward owing to the north–south compression (Fig. 1a)^2–4^. The North Anatolia Fault Zone (NAFZ) and East Anatolia Fault Zone (EAFZ) mark the northern and southeastern boundaries of the Anatolian Plate, respectively, and accommodate its westward motion. The NAFZ extends from the Karlıova triple junction (KTJ) to mainland Greece, with a total length of ~ 1,500 km and slip rate of ~ 20 mm/yr^5^. The NAFZ is characterized by right-lateral strike-slip movement along an almost east–west direction^6,7^. The EAFZ is a sinistral strike-slip fault that extends southwestward from the KTJ to the Amik Basin , with a total length of ~ 700 km and slip rate of ~ 9 mm/yr (Fig. 1)^8^. The fault intersects with the Dead Sea Fault Zone (DSFZ), which serves as the transform boundary between the African and Arabian plates^4,6,9^. Although both fault zones have experienced large destructive historical earthquakes^10^, the NAFZ had hosted 12 earthquakes of Mw ≥ 6.7 in the past century, while only one such earthquake (the 2020 Mw 6.8 Doğanyol–Sivrice earthquake) occurred along the EAFZ, indicating higher strain accumulation potential along the latter^2,11,12^.

The EAFZ has attracted significant attention owing to its relatively rapid slip rate^9,13–15^. Based on fault geometry, slip rate, and surface ruptures of historical earthquakes, Duman and Emre^16^ divided the EAFZ into seven segments from west to east: the Amanos, Pazarcık, Erkenek, Pütürge, Palu, Ilıca, and Karlıova segments. There are notable fault morphological differences between the eastern and western sections EAFZ, demarcated by the city of Çelikhan as a boundary, with significant differences observed in slip rate, and seismic activity. Specifically, the eastern section is characterized by a simple single fault with a strike of approximately 60°, while the western section form is formed with multiple sub-parallel secondary faults located north of the main EAFZ fault, resulting in more complex tectonic activity and slip partitioning. GNSS data indicate a stable slip rate of 9 mm/yr for the entire EAFZ which rapidly decreases to 4.5 mm/yr for the southwestern segment (Türkoğlu segment). Additionally intersecting with the EAFZ is the northern DSFZ, which exhibits a slip rate of 4.2 mm/yr^4,6,17^. During 2007–2020, seismic activity east of Çelikhan was linearly distributed along the main EAFZ fault; west of Çelikhan, activity was dispersed throughout the entire secondary fault zone^13^. The frequency of intermediate–large earthquakes is significantly lower in the western section compared to the eastern section, which is similar to the interseismic seismic activity pattern^2,12,15,18–20^. Over the past 200 years, the eastern section of the EAFZ has experienced six earthquakes of Mw ≥ 6. In contrast, only three such events have been recorded on different sub-faults of the western section of the EAFZ (the 1872 M7.2 earthquake on the Amanos segment, the 1893 M7.1 earthquake on the Erkenek segment, and 1986 Mw 6.0 earthquake on the northern branch of the EAFZ, known as the Sürgü Fault Zone). The lower occurrence of medium–large earthquakes in the western section of the EAFZ, as compared to the eastern section, is consistent with patterns of interseismic seismic activity^2,12,15,18–20^. The complexity of fault geometry and tectonic activity is higher in the western section of the EAFZ compared with the eastern section. However, during the instrumental observation period, there have been almost no intermediate–large earthquakes recorded in the western section, which hinders a detailed understanding of the kinematics of the western Section^15^. In particular, there is debate regarding the continuation of the southwestern segment (Türkoğlu). The most widely accepted viewpoint suggests that the EAFZ changes trend from east to west at Türkoğlu, which bends ~ 30° southward along the Amanos segment and intersects with the DSFZ in the Amik Basin. However, a second viewpoint proposes that the EAFZ intersects with the DSFZ at Türkoğlu and then continues westward, cutting through the Amanos Mountains and connecting with the Toprakal and Yumurtalık faults in Iskenderun Bay^9,15,16,21–25^.

On February 6, 2023, a destructive Mw 7.9 earthquake struck central–southern Türkiye and northern Syria. The United States Geological Survey (USGS) located the epicenter at ~ 37.226 °N and ~ 37.014 °E, with a focal depth of ~ 10 km. Approximately 9 h later, a Mw 7.7 earthquake occurred ~ 95 km north of the Mw 7.9 earthquake epicenter (~ 38.011 °N and ~ 37.196 °E) with a focal depth of ~ 7.4 km^26–32^. The first earthquake involved the Amanos, Pazarcık, and Erkenek segments of the western section of the EAFZ, while the second occurred on the SFZ (i.e., the northern branch fault of the EAFZ). Both earthquakes were characterized as left-lateral strike-slip events. Together, these earthquakes resulted in widespread damage and > 50,000 deaths^28^. Previous studies have primarily focused on the rupture characteristics of the earthquake doublet, interaction between faults, and causes of the resulting damage. Despite variation in magnitudes and slip distributions due to different data sources and inversion methods, the first-order features of magnitude and slip distribution are consistent among these studies, which suggest the presence of supershear ruptures. However, there are differences in the spatiotemporal distribution of these ruptures^27,31,33–36^. Meng et al.^37^ mapped surface offset data through rapid post-earthquake field investigation, and found that the maximum displacement of the first earthquake event was located at the intersection of the Narli fault and the Pazarcik segment of EAFZ. Zhang et al.^38^ analyzed the velocity evolution of different segments of the fault based on back-projection technology, and found that the geometric characteristics of the fault were an important factor affecting the occurrence of supershear. Liu et al.^39^ obtained a detailed rupture process through joint inversion of multiple sources of data. Both Liu et al.^39^ and Zhang et al.^38^ suggest that the rupture velocity of the first earthquake event was lower in the southwest segment, but increased to 3.8 km/s on the Amonas segment. The second earthquake event ruptured as supershear in the western segment and sub-shear in the eastern segment. Ren et al.^40^ mainly discussed the triggering mechanism of supershear rupture in the 2023 Türkiye–Syria earthquake, indicating that supershear rupture also occurred on the initial branch of the first earthquake event, and the dynamic stress of the initial branch rupture and the high prestress level in the Pazarcik segment jointly promoted the Pazarcik segment to enter supershear rupture. However, the cascade rupture mechanism of the 2023 Türkiye–Syria earthquake is still unclear. Based on geological and geomorphological investigations, accumulated geological offsets along the western section range from ~ 19 to 26 km, while the eastern section has accumulated offsets of ~ 9–22 km^16^. The onset time of the Amanos segment (> 5 Ma) is earlier than Pazarcık (3–5 Ma) and Erkenek (< 3 Ma) segments^41^. Following the studies conducted by Manighetti et al. ^42^ and Dolan and Haravitch ^43^, the maturity of faults can be classified by jointly considering the initiation age (I-Age) and net slip (DTotal) of the fault. Manighetti et al.^42^ classify faults into “mature faults” (I-Age ≥ 10 Ma and/or DTotal ≥ 100 km), “immature faults” (I-Age < a few Ma and/or DTotal < 10 km), and faults of “intermediate maturity” (5 < I-Age < 10 Ma and/or DTotal ~ a few tens of kilometers). Taking into account the initiation age and net slip of the fault involved in the Mw7.9 earthquake event, we consider the west of EAFZ to have low-moderate structural maturity. The occurrence of large earthquake with supershear ruptures is generally not associated with fault system that has relatively low–moderate structural maturity; therefore, this event has prompted the necessity to study such events in order to improve seismic hazard assessments. Moreover, given the absence of instrumental recordings of large-sized (M ≥ 6.5) earthquakes along the western section of the EAFZ over the past century, the 2023 earthquake doublet provides an excellent opportunity to gain deeper understanding of the kinematic characteristics of the EAFZ and the mechanisms of earthquake generation on this immature fault.

The aim of this research was to provide a comprehensive description of the seismogenic faults and coseismic slip distribution of the 2023 Türkiye–Syria earthquake doublet. Furthermore, we aimed to investigate the formation mechanism of large earthquakes on immature faults and explore the kinematic characteristics of the western section of the EAFZ. Additionally, we aimed to present a conceptual model that to discuss the debate regarding this fault system: the western development of the EAFZ beyond Türkoğlu. To achieve these goals, we used SAR data, strong motion data, and GNSS data to jointly constrain the surface deformation field. The acceleration data recorded by the strong motion stations are converted to displacement data using baseline correction techniques. For ALOS-2 data analysis, the displacement field is obtained based on D-InSAR technology. However, due to significant deformation gradients in the near field of the earthquake, the coherence of Sentinel-1 satellite data in the C-band is severely affected. To address this issue, we used Sentinel-1 data to obtain the displacement field based on pixel offset tracking (POT) technology. Subseguently, we combined GNSS data with the strong motion data to determine the displacement field caused by the earthquake doublet. Moreover, we used finite fault inversion methods to determine the kinematic characteristics of the seismogenic faults.

## Data and methods

### Strong motion data and processing

**Figure 2:**
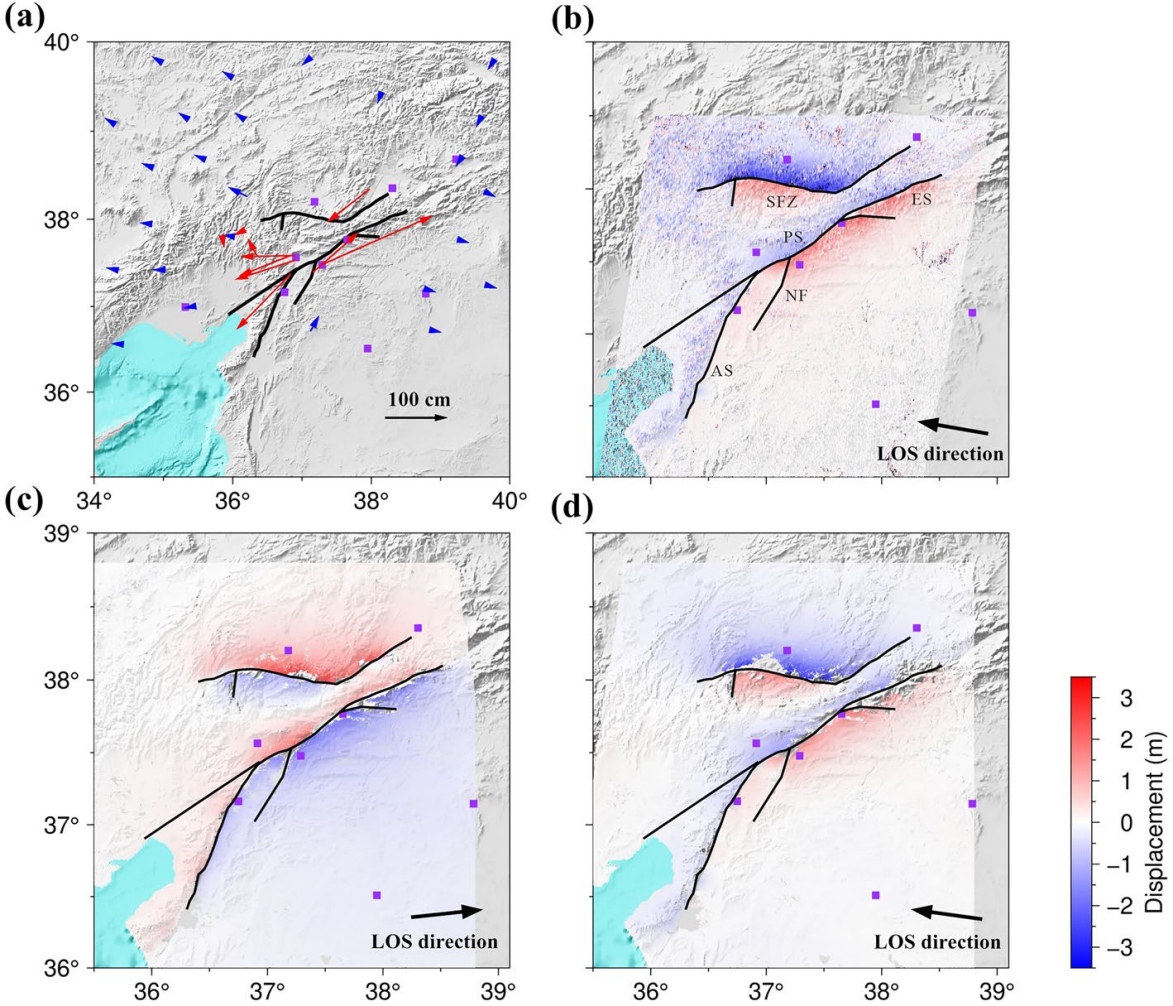
Coseismic displacement of the 2023 Türkiye–Syria earthquake doublet. (**a**) Coseismic horizontal displacement fields of the Mw 7.9 earthquake and Mw 7.7 earthquake. Red arrows indicate horizontal displacements obtained from strong motion data. Blue arrows represent coseismic horizontal displacements from GNSS data. (**b**) Coseismic range offset from Sentinel-1 satellite track D21. (**c**–**d**) Coseismic LOS displacements from ALOS-2 satellite from ascending track 184 and descending track 77. The black line represent the rupture faults of the 2023 Türkiye–Syria earthquake doublet. The purple rectangle represents the surrounding major cities. AS: Amanos segment; PS: Pazarcık segment; ES: Erkenek segment; NF: Narlı Fault; SFZ: Sürgü Fault Zone.

**Figure 3:**
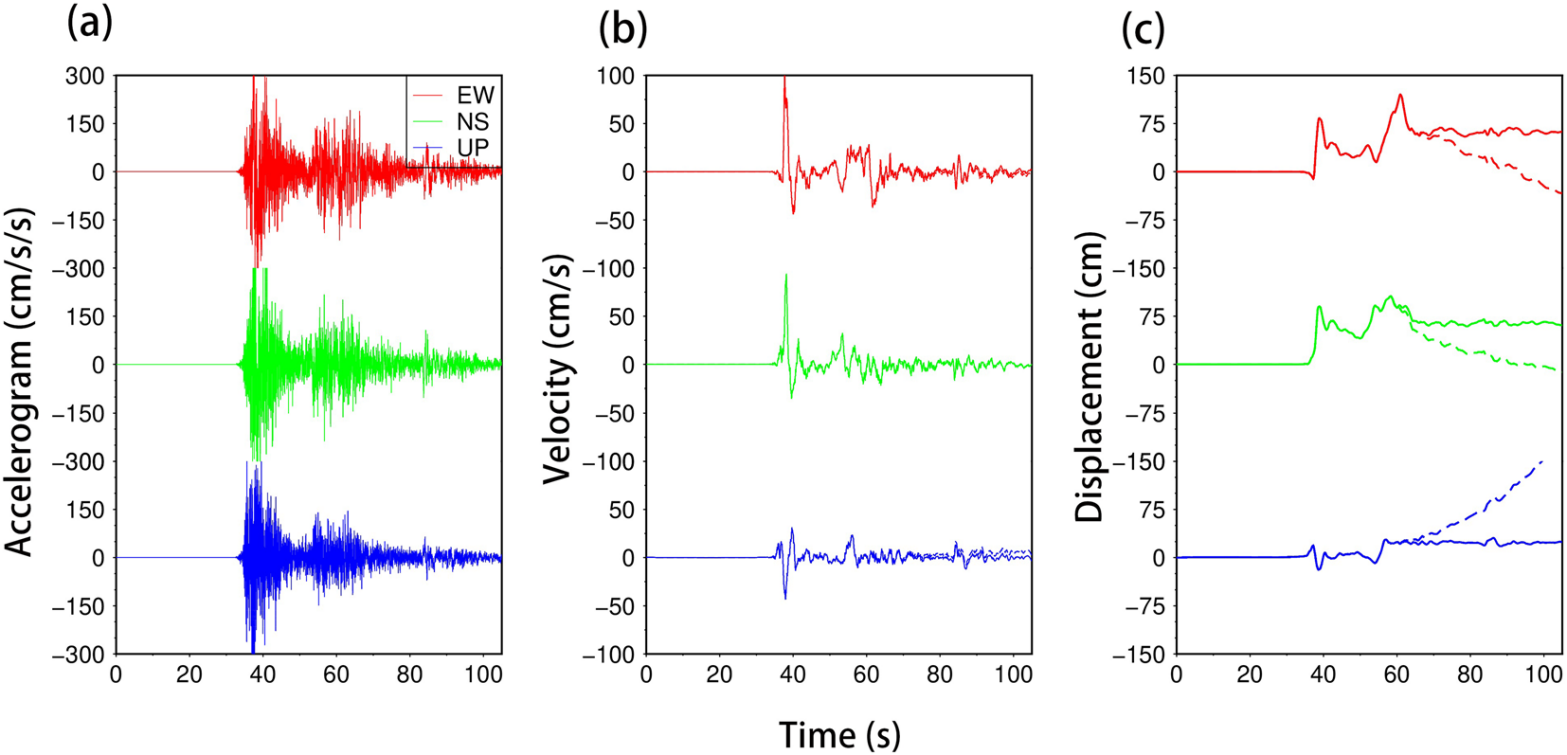
Baseline correction of strong motion data from station NAR. Uncorrected acceleration waveform (**a**), velocity waveform (**b**), and displacement waveform (**c**). Dotted lines show uncorrected data and solid lines show baseline-corrected data.

The Turkish Strong Motion Network is densely distributed near the EAFZ. Considering station–fault distance and data quality, we selected 75 acceleration components from 25 stations located within a 150 km radius of the Mw 7.9 earthquake’s epicenter, and 105 acceleration components from 35 stations situated within 200 km of the Mw 7.7 earthquake’s epicenter. These strong motion data suffer from significant baseline drift due to dynamic movements of rotation and translation making it impossible to obtain accurate displacement directly. Therefore, before integrating the acceleration data, we used the SMBLOC software to correct for the effect of baseline drift based on an automated empirical baseline correction method, and then performed double integration on the acceleration data to obtain stable displacement waveforms ^44^. In order to jointly invert the fault slip distribution with SAR data, we selected 12 stations that exhibited good displacement responses during both earthquakes. The displacement fields of both earthquakes exhibit a four-quadrant distribution (Fig. 2a), consistent with left-lateral strike-slip. For example, station NAR (located 20 km northeastern of the Mw 7.9 earthquake fault) primarily exhibited northeastward motion (Fig. 3), with eastward displacement of ~ 0.64 m, northward displacement of ~ 0.67 m, vertical displacement of ~ 0.23 m, and total static displacement of ~ 0.95 m.

### GNSS data and processing

The Continuously Operating Reference Station (TUSAGA Aktif: CORS-TR) and International GNSS Service (IGS) network provide a dense network of global navigation satellite system (GNSS) stations in the epicentral region. We obtained coseismic deformation fields for the two earthquakes using 198 three-component displacement data from 66 GNSS stations processed by Barbot et al. ^33^. In order to jointly invert the slip distribution with SAR data, and considering the quality of the data, we selected 26 stations that exhibited good data quality during both the Mw7.9 earthquake and the Mw7.7 earthquake events. Both displacement fields are consistent with left-lateral strike-slip motion (Fig. 2a). In summary, the strong motion and GNSS data are consistent in terms of magnitude and direction.

### SAR data and processing

Due to the high spatial resolution, accuracy, and all-weather capabilities of SAR data, it plays a crucial role in monitoring seismic deformation. Therefore, we utilized SAR data provided by the L-band ALOS-2 satellite operated by the Japan Aerospace Exploration Agency (JAXA) and the C-band Sentinel-1 satellite operated by the European Space Agency (ESA) to capture the surface displacement field of the 2023 Türkiye–Syria earthquake doublet. The magnitude of these earthquakes was significant, resulting in substantial deformation gradients in the near-field region. For the ALOS-2 data, ascending track 184 and descending track 77, which covered the seismic area well, were selected. The branch-cut method was employed for phase unwrapping, and the ionospheric noise influence was corrected by fitting a linear ramp to the far-field displacements. Finally, the surface displacement field caused by the earthquake was obtained (Fig. 2c, d). Regarding the Sentinel-1 data, descending track 21 was used. When obtaining the deformation field based on the D-InSAR technique using the C-band Sentinel-1 data, the large deformation gradients in the near-field region resulted in phase variations exceeding half a cycle between neighboring pixels and changes in scattering due to phenomena such as building collapses. Consequently, the near-field interferometric fringes were severely decorrelation, making it impossible to accurately retrieve the near-field deformation. Therefore, the pixel offset tracking technique was employed to capture the significant deformation caused by the 2023 Türkiye–Syria earthquake doublet ^45^. This technique is not affected by phase coherence and calculates displacements by registering two images. Firstly, the pre- and post-earthquake images of the 2023 Türkiye–Syria earthquake doublet were aligned, and then offsets were computed using a 64 × 192-pixel search window based on intensity correlation methods, as shown in Fig. 2b.

## Results

**Figure 4:**
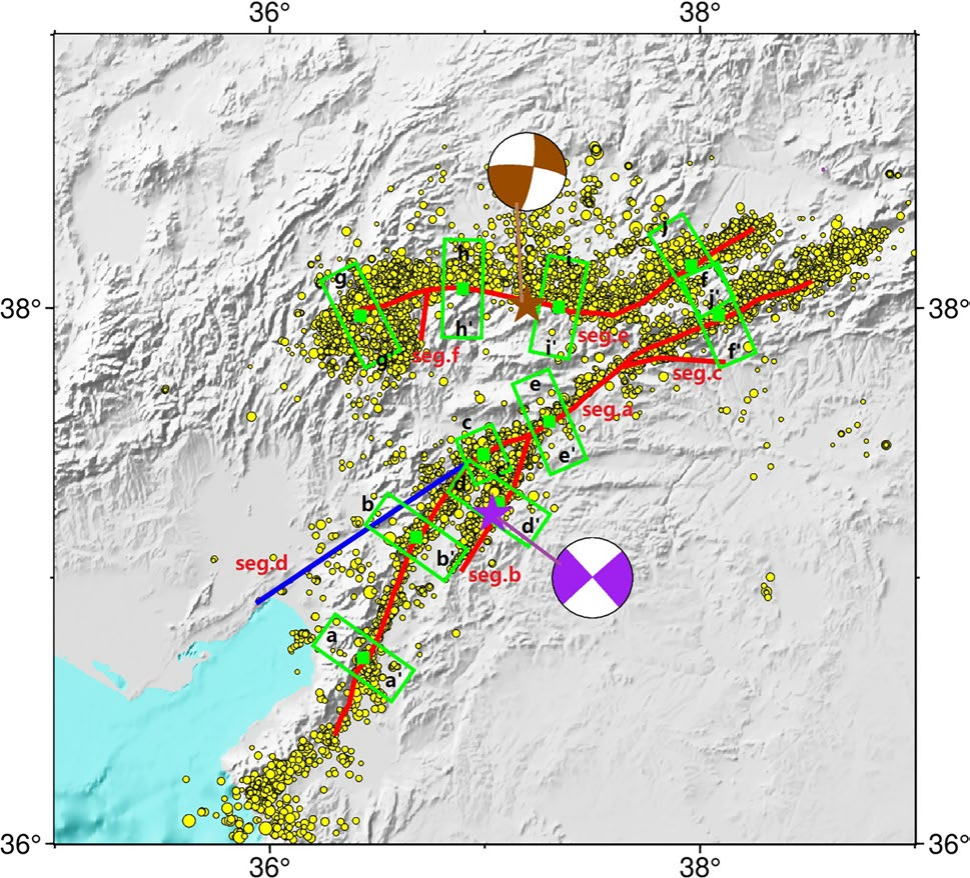
The geometry of fault models used for inversion of the 2023 Türkiye–Syria earthquake doublet. Red lines represent known faults used in all fault model inversions; the blue line represents fault-1, an uncertain fault that may have been involved in the Mw 7.9 earthquake. Green rectangular boxes indicate regions where aftershock projections were made for fault section analysis. The black letters indicate the direction of the section corresponding to the numbers in Fig. 5. The red numbers on the faults correspond to the numbers in Fig. 6. Magenta and brown stars represent the locations of the Mw 7.9 and Mw 7.7 earthquakes, respectively. Corresponding beach balls represent focal mechanism solutions.

**Figure 5:**
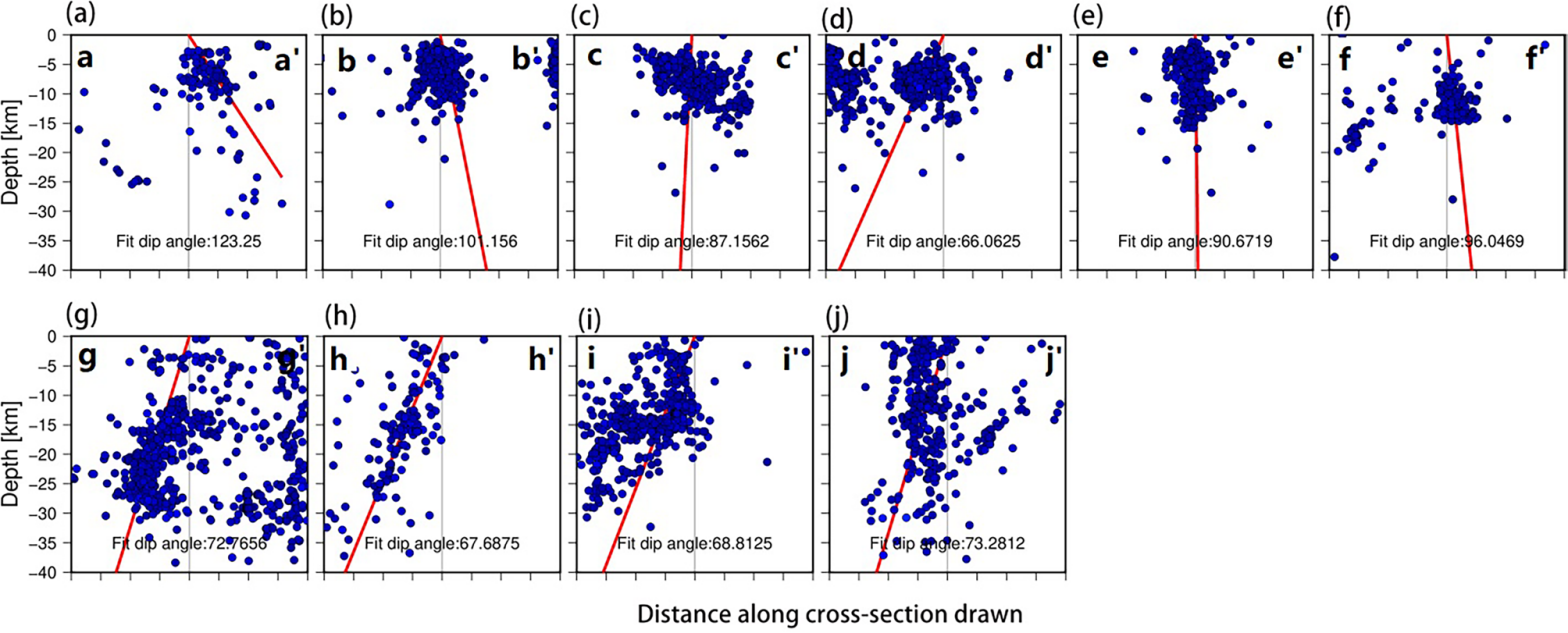
Projections of aftershocks of the 2023 Türkiye–Syria earthquake doublet on selected fault sections. Red lines represent the fault dip angles fitted to the distributed seismicity.

Before conducting coseismic slip distribution inversion, it was necessary to determine the geometric model of the seismogenic fault. The 2023 earthquakes caused significant surface ruptures, providing surface constraints for fault modeling ^26,34^. Based on field investigations and remote sensing data, both earthquakes involved multiple faults and cannot be approximated using a single planar fault. Surface rupture trace and the aftershock distribution indicate that the Mw 7.9 earthquake involved the Amanos, Pazarcık, and Erkenek segments of the EAFZ. Based on the surface rupture trace provided by the USGS as the surface constraint of the fault geometry, we use the aftershock fitted angle to determine the dip of the fault geometry. According to earthquake locations and previous studies, the Mw 7.9 earthquake nucleated on the secondary Narlı Fault (NF) ^31,34^. Due to ongoing debate regarding the development of the EAFZ west of Türkoğlu, it is uncertain whether the Mw 7.9 earthquake ruptured an unrecognized fault (referred to as ‘fault-1’, indicated by the blue line in Fig. 4). Therefore, we set two fault models: one comprising of the main fault and two branching faults, while the other consisting of the main fault, the two branching faults as well as the fault-1. To determine fault dip angles, we selected six locations along the fault trace and projected the aftershocks within a 16-km range along strike onto a cross-section (Fig. 5). The main fault was found to be south-dipping with a dip angle of 85 degrees; the NF was found to be north-dipping with a dip angle of 80 degrees. Preliminary determination based on aftershock locations suggests that the Mw 7.7 earthquake occurred on the SFZ. Therefore, we set the fault geometry of the Mw 7.7 earthquake to consist of two segments and selected four locations on the main fault to project the aftershocks. We found that the fault has a tendency of northward dip with an approximate dip angle of 80 degrees. The upper boundary of the fault geometry for both earthquakes was located at the surface, with a width of 30 km in the downdip direction. We divided the fault models in sub-faults of 5 × 5 km. According to USGS–National Earthquake Information Center (NEIC) focal mechanism solutions ^29^, both faults are left-lateral strike-slip faults; therefore, we limited the rake to between − 90 and 90°.

Based on the Okada elastic half-space dislocation model^46^, once a fault model is determined, the only unknown parameters are the slip amounts of each sub-fault. Therefore, there is a linear relationship between fault parameters and observed surface deformation values, which can be expressed as:

1 $${\text{d}}={\text{G}}\times {\text{m}}+\upvarepsilon$$

where d is observed surface displacement, m denotes the fault parameters associated with each sub-fault, and $\upvarepsilon$ is the error related to the observations and model construction. To obtain stable and reliable solutions by avoiding ill-conditioning problems in the linear equations, a smooth constraint is applied to the slip distribution, ensuring an appropriate roughness of the model’s slip distribution. Thus, the inversion problem for slip distribution can be transformed into a problem of minimizing an objective function, which can be expressed as:

2 $${\text{F}}({\text{m}})={\Vert {\text{Gm}}-{\text{d}}\Vert }^{2}+{\mathrm{\alpha }}^{2}{\Vert {\text{Hm}}\Vert }^{2}$$

where $\mathrm{\alpha }$ is the smoothing factor and ${\text{H}}$ is the Laplacian operator. In this study, the steepest descent method was used to solve the slip distribution^44^. Given the varying uncertainties associated with different datasets, we assign different weights to each dataset during the inversion process. The vertical component of GNSS data is known to have lower accuracy compared to the horizontal component; thus, we assign it a relatively lower weight. On the other hand, the D-InSAR results of ALOS-2 data exhibit higher precision than the POT result from Sentinel-1 data, so we assign it a relatively higher weight. Through a trial-and-error approach, we ultimately set the weights for ALOS-2, strong motion, horizontal GNSS, Sentinel-1 data, and vertical GNSS as 5:5:5:3:2. To determine the optimal smoothing factor, we conducted a grid search with a step size of 0.01 within the range of 0–0.3 to search for the best smoothing factor. Subsequently, we selected the values of misfit and roughness at the maximum inflection point and ultimately determined the optimal smoothing factor was 0.03 (Fig. S5).

**Figure 6:**
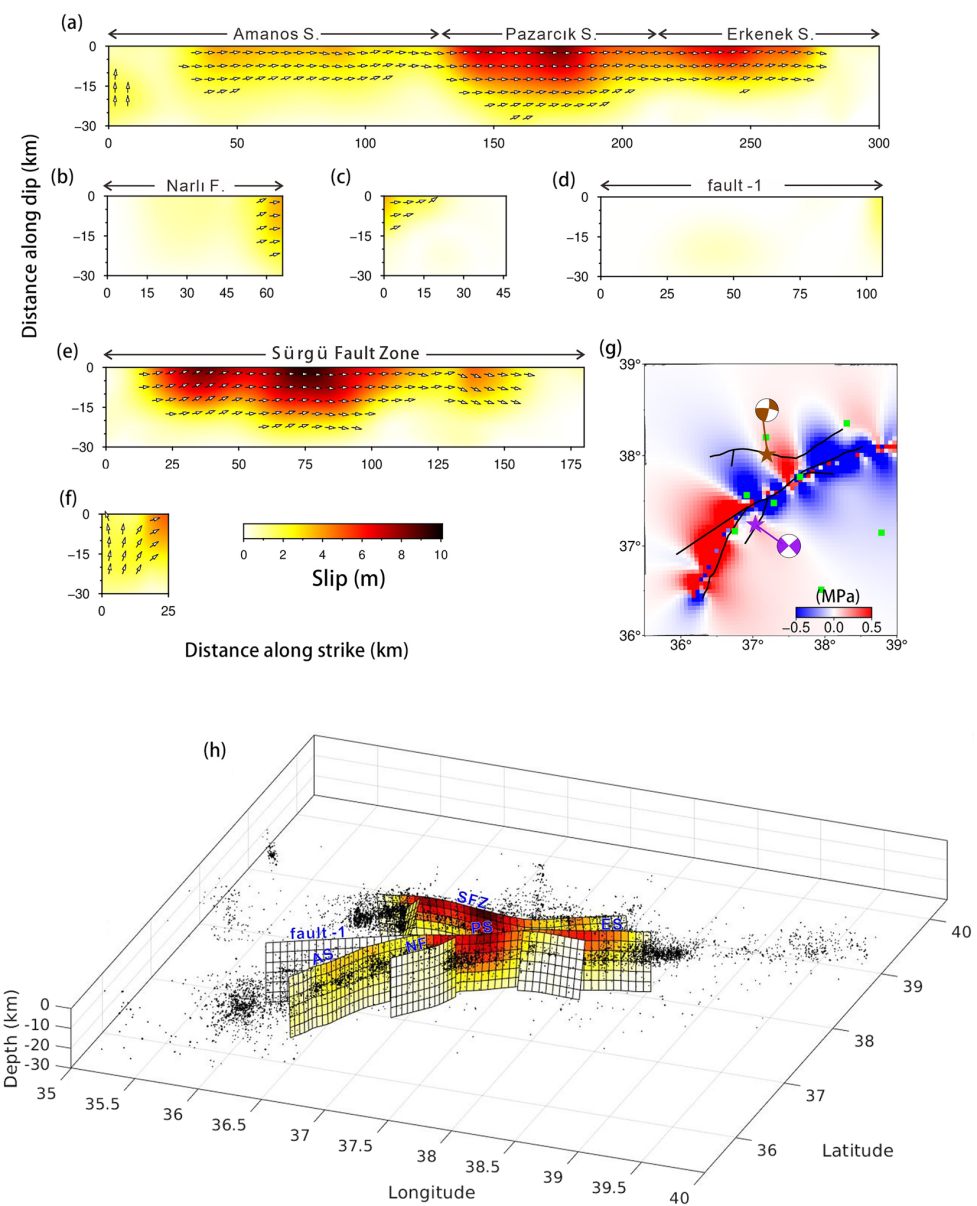
The co-seismic slip distribution model of the 2023 Türkiye–Syria earthquake doublet. (**a**–**d**) Co-seismic slip distribution of the Mw 7.9 earthquake event, where (**a**) represents the main fault along the EAFZ, (b–d) represents three bifurcate fault from west to east, and (**e**–**f**) show the co-seismic slip distribution of the Mw 7.7 earthquake event, where (**e**) represents the Sürgü Fault Zone. (**g**) The Coulomb stress changes induced by the Mw7.9 earthquake event at a depth of 10 km. The receiving fault is oriented in the east–west direction; the green filled boxes indicate the positions of major cities near the epicenter. (**h**) Three-dimensional schematic diagram of co-seismic slip and aftershocks. Fault numbers correspond to those in Fig. 4.

The co-seismic slip inversion results of the Mw 7.9 earthquake, based on SAR, strong motion and GNSS data, reveal three asperities on the main fault (Fig. 6a–d). After conducting the checkboard test, our used data provides strong constraints on the set fault geometry within a depth of 15 km. Our findings indicate that the coseismic slip of the 2023 Türkiye–Syria earthquake mainly occurred within 15 km, leading us to believe that our results are quite reliable (Fig. S6). The westernmost asperity, located on the Amanos segment, had a maximum slip value of 4 m, primarily distributed within a depth of 15 km. The middle asperity had a maximum slip value of ~ 7 m, mainly concentrated within a depth of 20 km. The eastern asperity had a maximum slip value of ~ 5 m, primarily concentrated within a depth of 15 km. The maximum slip value on fault-1 was 1.5 m, mainly concentrated at depths of 0–20 km, where the fault-1 connects to the main fault; a lack of surface rupture is consistent with field investigations. The motion of all segments within 15 km depth was primarily left-lateral strike-slip, with a locally existing thrust component possibly related to the complex fault geometry. The seismic moment released by this earthquake event was equivalent to a Mw 7.85 event, which is similar to the USGS and Global Centroid–Moment-Tensor (GCMT) results (i.e., Mw 7.9). Meanwhile, the Mw 7.7 earthquake event had three asperities (Fig. 6e–f), the major asperity was located on the Çardak fault, with a high slip value of 9.5 m primarily concentrated within a depth of 25 km. The motion was almost pure left-lateral strike-slip, and the seismic moment released was equivalent to a Mw 7.67 earthquake event. The epicenter of the second event is located in the stress loading area of the first event (Fig. 6g), where the Coulomb stress loading exceeds 0.1 MPa and approaches the threshold for coseismic stress triggering. Therefore, we conclude that the Mw 7.7 earthquake was triggered by the static stress imparted by the Mw 7.9 earthquake.

The co-seismic slip of both earthquakes was shallow and concentrated, which may be an important factor contributing to the significant destructive impacts. Overall, our results are similar to the inversion results from other researches^29,32,34^. Figure 6h illustrates the geometric relationships between co-seismic slip and aftershocks for both earthquakes; aftershocks were mainly concentrated at the end of two seismogenic fault, suggesting stress loading in the unruptured area at the boundary of the fault after co-seismic rupture. As shown in Fig. S3 and S4, data fitting for both earthquakes was satisfactory, thereby confirming the reliability of our inversion results.

## Discussion

In recent years, the investigation of mega-earthquakes and their fault rupture behavior has emerged as a prominent research area. The 1911 Ms 8.0 Kenbin (Chon–Kemin) earthquake in Kyrgyzstan and Kazakhstan^47^, the 2008 Mw 7.9 Wenchuan earthquake in China^48^, the 2010 Mw 7.2 El Mayor-Cucapah earthquake in Mexico^49^, and the 2016 Mw 7.8 Kaikōura earthquake in New Zealand^50^ have all exhibited complex multifault ruptures, propagating through geometric boundaries of fault segments such as the stepovers or bending structure, challenging previous understandings regarding structural zoning, fault activity, fault segmentation and seismic hazard assessment. The Türkiye–Syria earthquake doublet on February 6, 2023 stands as the largest and deadliest seismic event in the region over the past century. The two strong earthquakes took place on the main and northern strands of the EAFZ, which are connected by a complex network of faults. According to previous geological and geomorphological studies, the western section of the EAFZ is classified as a young–moderately mature fault zone with a cumulative offset of 19–26 km. The occurrence of such large earthquakes on such an immature fault is still difficult to understand^16,51^. The EAFZ consists of seven segments (Table 1) connected by bends or stepovers of different sizes. The historical earthquakes and seismic events recorded by instruments on the EAFZ have predominantly been confined to individual segments, suggesting that bends and stepovers on the EAFZ generally serve as barriers to rupture propagation.

**Table 1:** Geometric, kinematic, and dimensional characteristics of segments within the EAFZ and their related fault jogs (modified after Duman and Emre^16^).

Strand of the EAFZ	Fault segment			Fault jog	
	Name	Strike	Length (km)	Name	Type
Main strand	Amanos	N35 °E	112	Türkoğlu	Releasing bend
	Pazarcık	N60 °E	82		
	Erkenek	N75 °E	62	Gölbaşı	Releasing stepover
	Pütürge	N60 °E	96	Yarpuzlu	Restraining double bend
	Palu	N62 °E	77	Lake Hazar	Releasing bend
	llıca	N40 °E	40	Gökdere	Restraining bend
	Karlıova	N50 °E	31	Göynük	Paired bend
Northern strand	Sürgü	EW	55	Nurhak	Fault complexity
	Çardak	EW	85		
	Savrun	N50 °E	60	Göksun	Releasing bend

The Mw 7.9 earthquake ruptured three segments on the EAFZ—the Amanos, Pazarcık, and Erkenek segments—which are connected by the Türkoğlu releasing bend and Gölbaşı releasing stepover. In addition, there are several stepovers within the segments. Based on finite fault inversion, the co-seismic slip distribution of the Mw 7.9 earthquake included three main asperities. The western asperity on the southwestern Amanos segment demonstrated a high slip value of up to 4 m, which is consistent with the high strain rate location observed in the GNSS-derived strain rate field. The central asperity was located on the junction of the NF and the EAFZ, while the Türkoğlu releasing bend is located at the junction of the Amanos and Pazarcık segments. Meanwhile, the eastern asperity extended across the junction of the Pazarcık and Erkenek segments (Gölbaşı Releasing stepover), and a stepover within the Erkenek segment. The positional consistency between the two main asperities and fault bends/stepovers indicates that during seismic events before the 2023 Mw 7.9 earthquake, rupture propagation stopped at the fault jog, resulting in the continuous accumulation of stress at these barriers. The Mw 7.9 earthquake nucleated on the NF, and then propagated northeastward along the EAFZ, triggering rupture on the southwest side of the junction between the NF and EAFZ^31^. When the rupture on the NF propagated to the Pazarcık segment, it temporarily stopped; however, within a few seconds, Coulomb stress change caused by slip on the NF triggered rupture within the Pazarcık segment, which propagated northeastward along the EAFZ, breaking through the barrier formed by the Gölbaşı releasing stepover and a stepover within the Erkenek segment. In this process, Coulomb stress change caused by slip on the NF and the asperity on the eastern Pazarcık segment triggered rupture on the southwestern segment at the junction with the NF and EAFZ. This rupture broke through the barrier formed by the Türkoğlu releasing bend, and continued to propagate along the Amanos segment, triggering slip on the fault-1. This suggests that high magnitude earthquakes on immature faults are largely due to the geometric complexity of bends and stepovers, which lead to cascading rupture events. When the energy released by a rupture within a segment is not sufficient to break through barriers formed at the boundaries, stress accumulates at the segment boundaries. After several earthquake cycles, when stress on multiple adjacent barriers has accumulated to a certain extent, cascading ruptures may occur, resulting in a larger and more destructive earthquake event. Zhang et al.^38^ found that on simple fault plane structures, supershear ruptures are more likely to have occurred, leading to a sudden release of stress and the formation of higher slip gradients at the barriers. It futher facilitates the generation of cascading ruptures. This suggests that the barriers played an important role in promoting the occurrence of cascading ruptures to some extent. Ren et al.^40^ proposed that the higher level of prestress on Pazarcık segment is a significant factor in generating supershear ruptures. The views from the aforementioned studies also support the conclusion of this study: bends and stepovers on moderately mature faults accumulate higher stresses over multiple seismic cycles, making them prone to sequential rupture in a cascading rupture during a single earthquake event.

**Figure 7:**
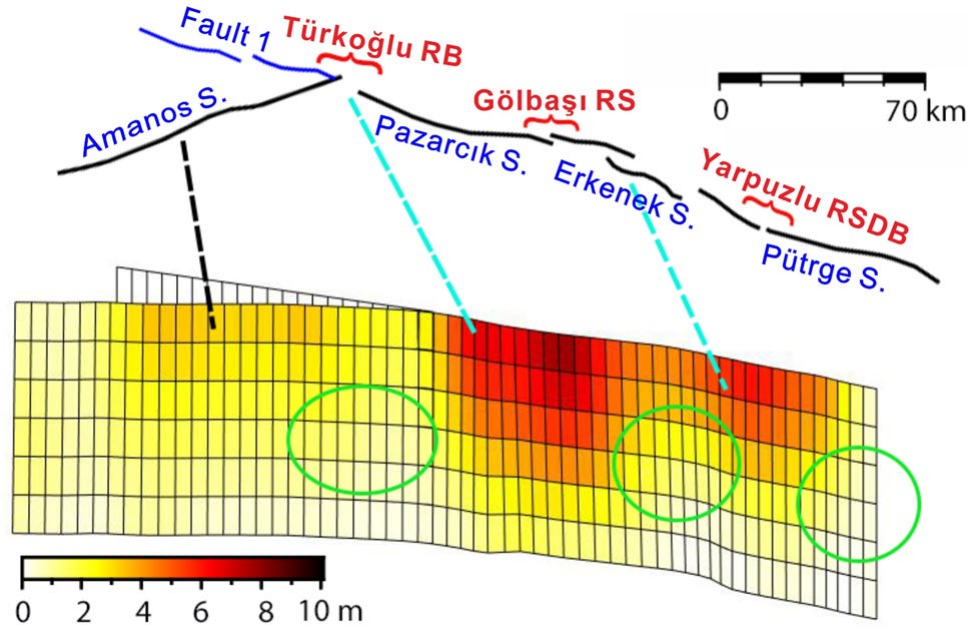
Schematic diagram of the seismic incubation mechanism for cascading ruptures on the immature fault. The black line indicates the surface trace on the west side of the EAFZ and the red brackets mark the locations of stepovers and bends on the fault zone. The light blue dashed lines mark the location of stepover or bend corresponding to the major asperity of the Mw7.9 seismic event. The black dashed line marks the southwestern Amanos segment corresponding to the western asperity demonstrated a high slip value of up to 4 m. The green ovals represent seismic events that occurred within the segments at different times.

In short seismic cycles, the presence of bends and stepovers inhibits rupture propagation, confining earthquakes within individual segments and leading to small–moderate seismic events (Fig. 7). The occurrence of stress loading on segment boundaries, specifically at locations with stepovers/bends, is attributed to the rupture of these smaller to moderate seismic events. However, during longer seismic cycles, barriers formed at bends and stepovers may develop futher and trigger cascading ruptures, resulting in larger earthquakes. This phenomenon could be a significant contributing factor behind the occurrence of large earthquakes on immature faults. In conclusion, we propose that the 2023 Mw 7.9 earthquake on the young–intermediate mature EAFZ can be attributed to complex fault geometry causing cascading ruptures of multiple asperities, localized high strain rates, and instantaneous supershear rupture.

According to geological and geomorphological studies, there are significant morphological differences between the eastern and western sections of the EAFZ, with Çelikhan as the boundary. The eastern section exhibits a linear single simple fault, while the western section is composed of multiple sub-parallel secondary faults. Interseismic seismic activity along the EAFZ is concentrated along the eastern sectiont, while seismic activity along the western section is more dispersed. Over the past two centuries, moderate to large earthquakes in the western section have been significantly fewer compared to that on the eastern section, indicating a complex stress partitioning pattern of the western section. Currently, many studies have investigated the slip rate of the EAFZ based on GNSS data. The generally accepted understanding is that the eastern EAFZ, east of Türkoğlu, has a stable slip rate of ~ 9 mm/yr, which rapidly decreases to 4.5 mm/yr on the Amanos segment west of Türkoğlu^4,6,17^. Owing to a lack of stations near the fault, it is difficult to obtain a more detailed slip rate distribution. Based on geological offsets, Duman and Emre ^16^ estimated a slip rate of ~ 8.3 mm/yr between Çelikhan and Karlıova, which is consistent with the results from geodetic measurements. However, their estimated slip rates west of Çelikhan differ to those based on geodetic measurements. The slip rate of the Erkenek segment was estimated to be 6.5–7.0 mm/yr, that of the Pazarcık segment was estimated to be 4.0–4.6 mm/yr, and those of the parallel overlapping Amanos and Yesemek segments were estimated to be 2.8 and 2.68 mm/yr, respectively. In contrast, geodetic data yield an approximate slip rate of 4.5 mm/yr for the Amanos segment, but are unable to distinguish between the Amanos and Yesemek segments. The difference in slip rates on either side of Çelikhan and the high strain accumulation released during the 2023 Mw 7.7 earthquake suggest the presence of slip partitioning along the EAFZ at Çelikhan. The slip rate of the SFZ is approximately 2 mm/yr^16^.

The EAFZ west of Türkoğlu remains a subject of debate. The dominant view postulates that the EAFZ west of Türkoğlu undergoes an ~ 30° bend towards the southwest, and continues along the Amanos segment with a strike direction of ~ 206°. However, an alternative view is that the EAFZ does not change its angle of development, and instead cuts directly across Amanos Mountains via fault-1, which extends along a strike direction of 236°^4,15,17,21,52^. Yönlü et al. ^15^ demonstrated linear seismic activity between Türkoğlu and the Yumurtalık fault during the interseismic period; however, seismic activity near Türkoğlu is rare, which supports the presence of fault-1. In this study, the model including fault-1 showed 1.5 m of coseismic slip on fault-1 (Fig. 6d), mainly concentrated at eastern end of the fault-1, where it connects with the main fault. No significant slip has been observed elsewhere along the fault. Therefore, we suggest that there was no noticeable slip on the fault-1 during the 2023 Türkiye–Syria earthquake doublet. Given the complexity of rupture propagation, the absence of the fault-1 involvement in this earthquake does not indicate that the EAFZ does not extend westward along the fault-1. Possible reasons for this could be the relatively low slip rate of the fault-1, which may not have accumulated enough strain or the rupture could not break through the barrier formed by the triple junction here and furthe prevented the slip from continuing to propagate westward. Further geodetic evidence is required to ascertain that the EAFZ bifurcates at Türkoğlu, and that slip partitioning occurred along the Amanos segment and fault-1.

## Conclusion

According to the results of coseismic slip inversion, the initial Mw 7.9 earthquake was primarily characterized by three asperities. The westernmost asperity had a maximum slip of 4 m, with slip mainly concentrated at depths of up to 15 km. The central and eastern asperities exhibited maximum slip values of 7 and 5 m, respectively, primarily at depths up to 20 km. A comparison of two fault geometric models revealed the rupture of a previously undetected fault, despite the absence of surface rupture. The slip was predominantly concentrated at depths ranging from 6 to 20 km, and the seismic moment release corresponded to an event with a magnitude of Mw 7.85. During the second (Mw 7.7) earthquake featured a main asperity situated on the Çardak fault, with a high slip value of 9.5 m primarily at depths up to 24 km.

The EAFZ is characterized by low–intermediate structural maturity, and rupture propagation and stress accumulation have occurred at bends and stepovers along the fault zone in previous seismic cycles. Stress release during the highly destructive 2023 Türkiye–Syria earthquake doublet overcame these barriers, resulting in cascading multi-segmented rupture. These findings have significant implication of hazard management in this region, and along other immature fault systems.

### Supplementary Information

Supplementary Figures.

## Data Availability

The hypocenter and surface rupture trace of 2023 Türkiye–Syria earthquake doublet were provided by the U.S. Geological Survey (https://earthquake.usgs.gov). Strong motion data were downloaded from the Department of Earthquake, Disaster and Emergency Management Authority (AFAD) of Türkiye (https://deprem.afad.gov.tr/). All websites were last accessed on 15 August 2023.
