# Supplementary material for: Immature characteristics of the East Anatolian Fault Zone from SAR, GNSS and strong motion data of the 2023 Türkiye–Syria earthquake doublet
Source: Sci Rep. 2024 May 9;14:10625. doi: 10.1038/s41598-024-61326-6 (PMC11082214; doi:10.1038/s41598-024-61326-6)
Supplement: Supplementary file 1 — Supplementary Figures. [file 41598_2024_61326_MOESM1_ESM.doc]

Supplementary Materials for “Immature characteristics of the East Anatolian Fault Zone from SAR, GNSS and strong motion data of the 2023 Türkiye–Syria earthquake doublet”

Jiao Liu^1, 2^, Chuanchao Huang^1*^, Guohong Zhang^1,2,3^, Xinjian Shan^1^, **Andrey Korzhenkov^4^**, Tuncay Taymaz^5^

^1^ State Key Laboratory of Earthquake Dynamics, Institute of Geology, China Earthquake Administration, Beijing 100029, China; [liujiao@ies.ac.cn](mailto:liujiao@ies.ac.cn) (J.L.); [cchuang@ies.ac.cn](mailto:cchuang@ies.ac.cn) (C.H.); [zhanggh@ies.ac.cn](mailto:zhanggh@ies.ac.cn) (G.Z.); [xjshan@163.com](mailto:xjshan@163.com) (X.S.)

^2^ Urumqi Institute of Central Asia Earthquake, China Earthquake Administration, Urumqi 830011, China; [zhanggh@ies.ac.cn](mailto:zhanggh@ies.ac.cn) (G.Z.); [liujiao@ies.ac.cn](mailto:liujiao@ies.ac.cn) (J.L.);

^3^ Institute of Disaster Prevention, Sanhe City, Hebei Province 065201, China; [zhanggh@ies.ac.cn](mailto:zhanggh@ies.ac.cn) (G.Z.)

^4^ Institute of Physics of the Earth, Russian Academy of Sciences, Moscow 123242, Russia; [korzhenkov@ifz.ru](mailto:korzhenkov@ifz.ru) (A.K.)

^5^ Istanbul Technical University, Ayazağa Campus, Maslak, Istanbul 34469, Türkiye; [ttaymaz@gmail.com](mailto:ttaymaz@gmail.com) (T.T.)

*Corresponding author: cchuang@ies.ac.cn

Institute of Geology, China Earthquake Administration, Yard No. 1, Hua Yan Li, Chaoyang District, Beijing 100029, China

**This supplement file includes:**

Figures S1 to S6

**
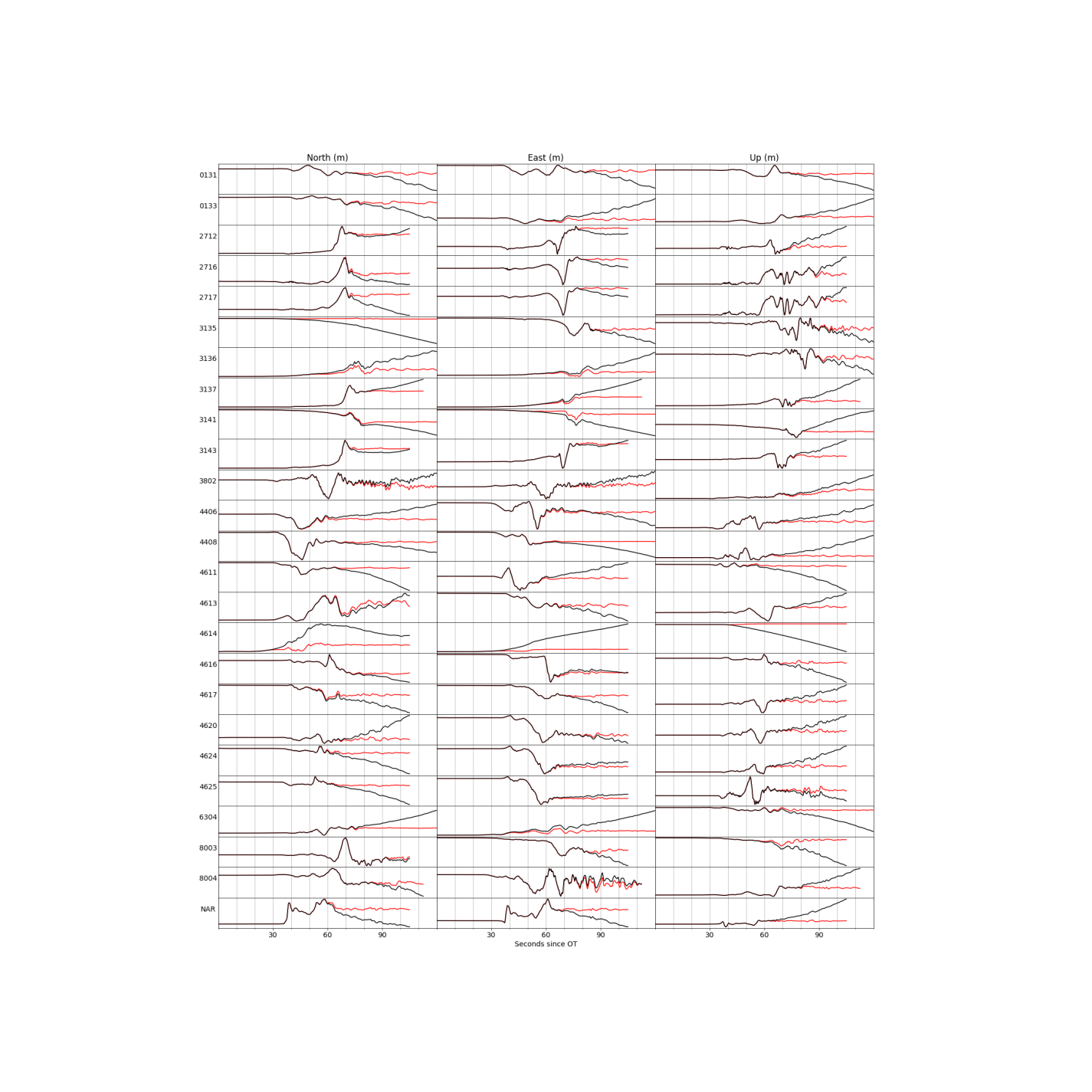
**

Figure S1. Baseline correction effect of data from 25 strong motion stations during the 2023 Türkiye–Syria Mw7.9 earthquake. The displacement waveform obtained from the strong motion data after baseline correction is compared with that from the strong motion data without baseline correction. The ordinate is the name of the station, and the abscissa indicates the duration since the rupture of the earthquake. The red line represents the displacement waveform obtained by integrating the strong motion data after baseline correction twice, while the black line represents the displacement waveform obtained by the strong motion data without baseline correction.


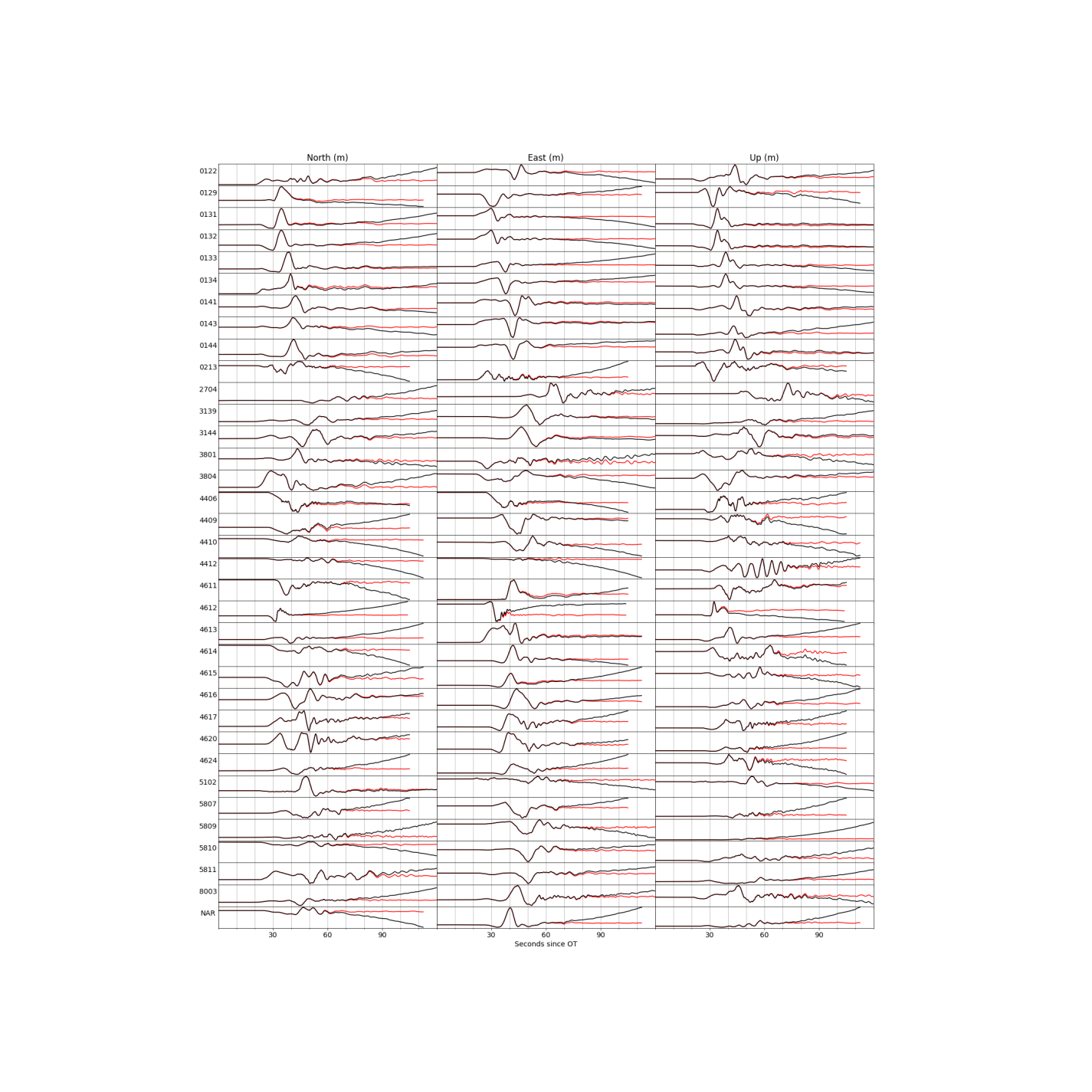


Figure S2. Baseline correction effect of data from 35 strong motion stations during the 2023 Türkiye–Syria Mw7.7 earthquake. The displacement waveform obtained from the strong motion data after baseline correction is compared with that from the strong motion data without baseline correction. The ordinate is the name of the station, and the abscissa indicates the duration since the rupture of the earthquake. The red line represents the displacement waveform obtained by integrating the strong motion data after baseline correction twice, while the black line represents the displacement waveform obtained by the strong motion data without baseline correction.


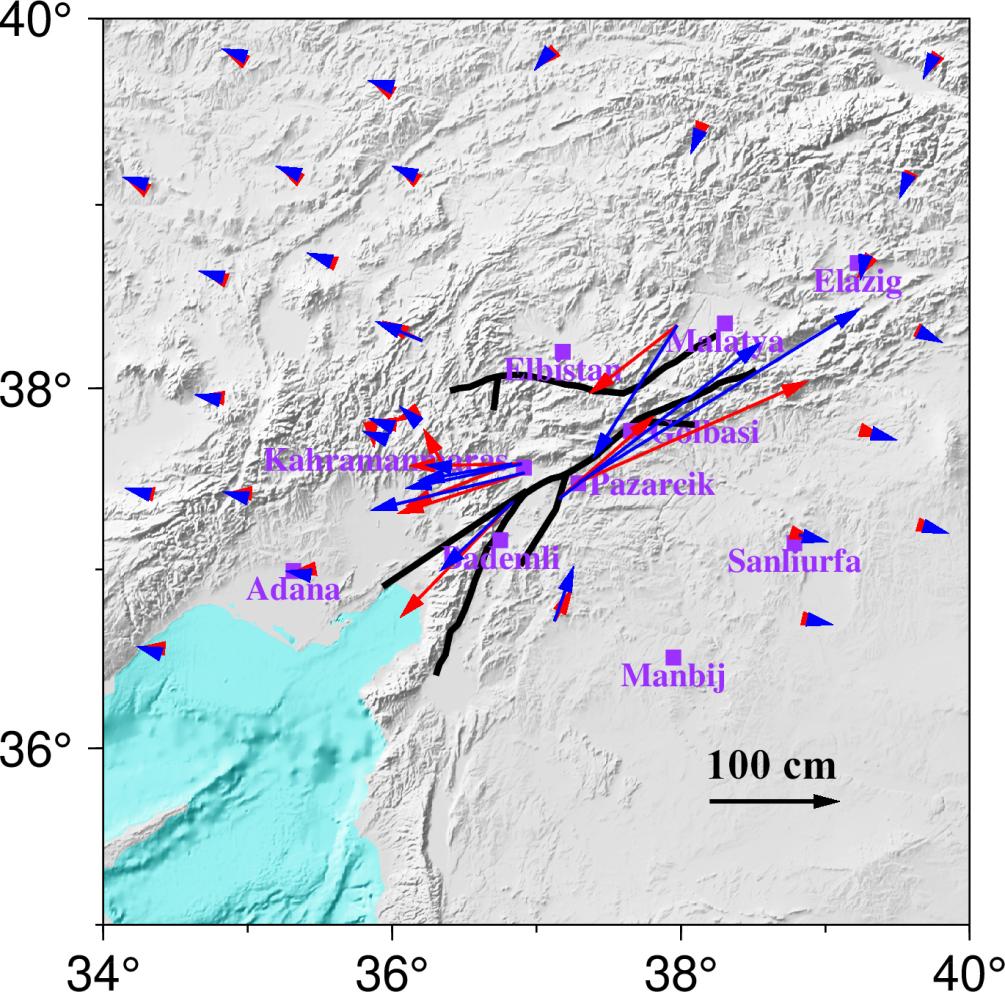


Figure S3. Data fitting for the inversion of the 2023 Türkiye–Syria earthquake doublet. Red arrows represent observed data and blue arrows represent simulated data based on the optimal slip model.


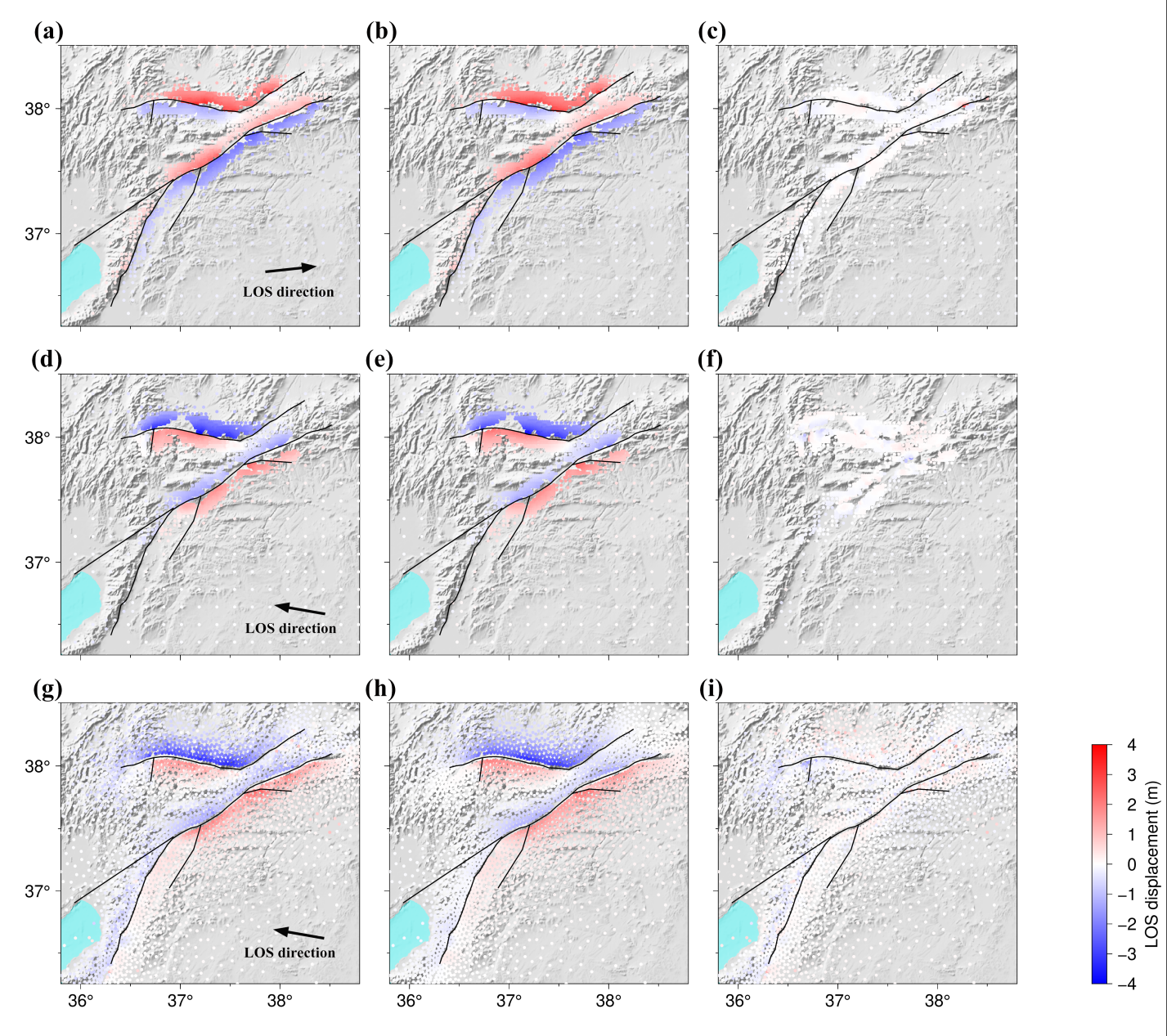


Figure S4. Observation, simulation, and residual of SAR data for the 2023 Türkiye–Syria earthquake doublet. The first column represents the observed data, the second column represents the simulated data based on the optimal slip distribution, and the third column represents the residual between the observed and simulated data. (a-c) represent the D-InSAR result of ALOS-2 Ascending track 184. (d-f) represent the D-InSAR result of ALOS-2 descending track 77. (g-i) represent the pixel offset tracking (POT) result of Sentinel-1 descending track 21.


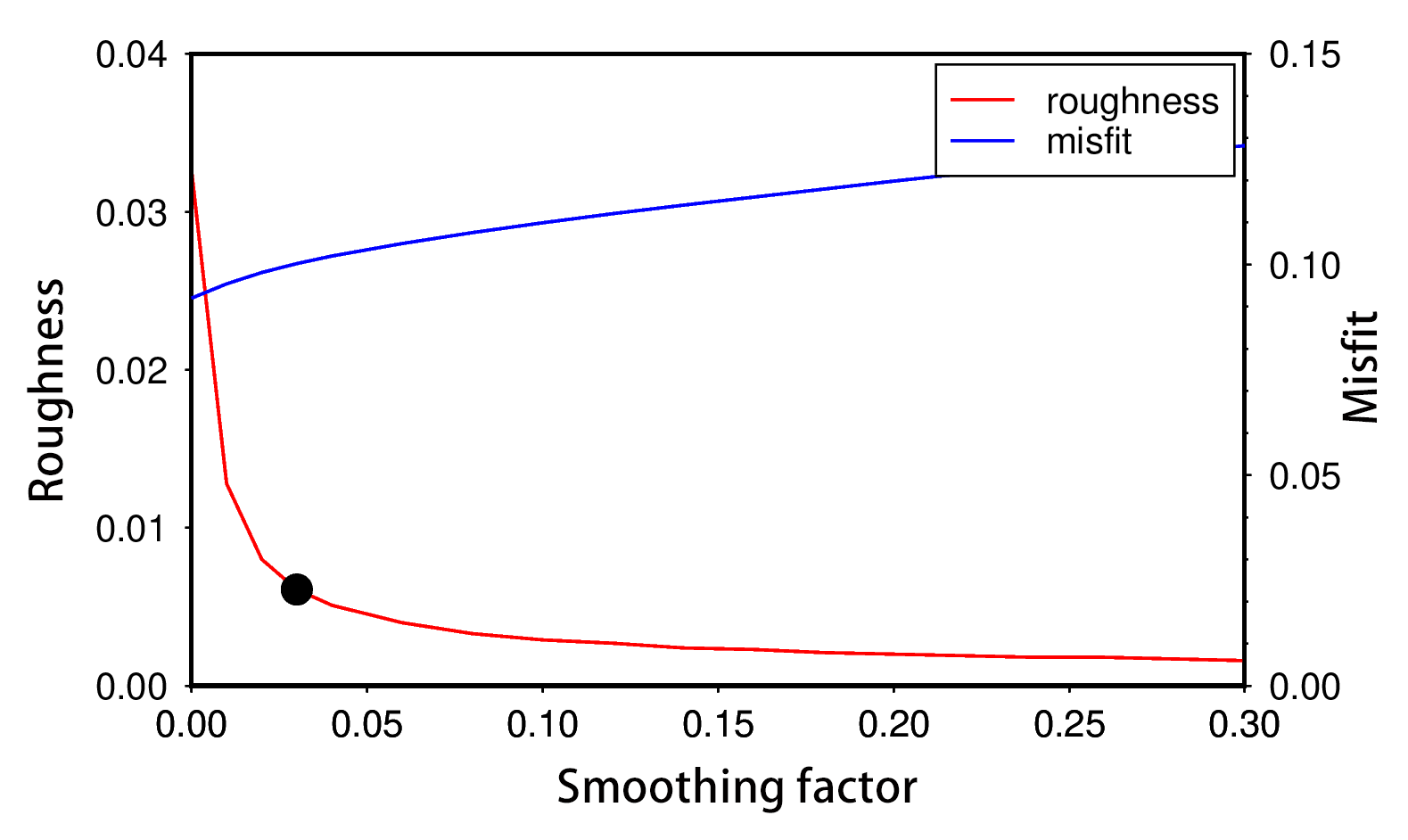


Figure S5. The roughness and misfit vary with the smoothing factor. The black circle indicates the position of the optimal smoothing factor.


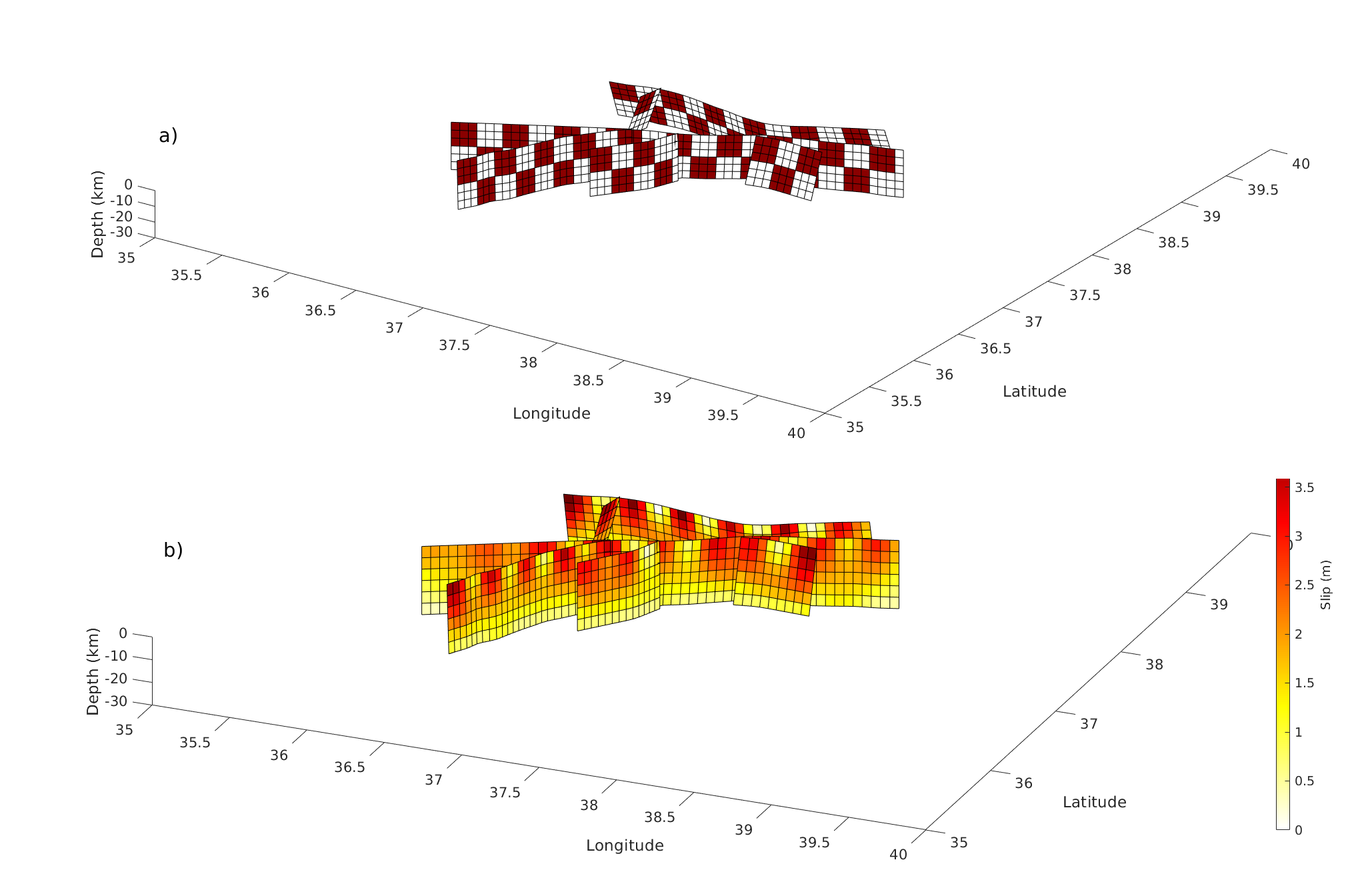


Figure S6. Checkboard test. (**a**) represents the assumed slip distribution, (**b**) shows the result of inverting observed data using the slip distribution from (**a**), with the same inversion parameters used in the inversion of the 2023 Türkiye–Syria earthquake doublet.
